# Tea and Coffee Consumption and Risk of Laryngeal Cancer: A Systematic Review Meta-Analysis

**DOI:** 10.1371/journal.pone.0112006

**Published:** 2014-12-12

**Authors:** Jiangbo Chen, Shuo Long

**Affiliations:** Department of Otolaryngology, Central South University Affiliated The Third Xiangya Hospital, Changsha, Hunan, China; University of Tennessee, United States of America

## Abstract

**Background:**

Tea and coffee are the most commonly consumed beverages in the worldwide. The relationship between tea and coffee consumption on the risk of laryngeal cancer was still unclear.

**Methods:**

Relevant studies were identified by searching electronic database (Medline and EMBASE) and reviewing the reference lists of relevant articles until Oct. 2013. Observational studies that reported RRs and 95% CIs for the link of tea and coffee consumption on the risk of laryngeal cancer were eligible. A meta-analysis was obtained to combine study-specific RRs with a random-effects model.

**Results:**

A total of 2,803 cases and 503,234 controls in 10 independent studies were identified. The overall analysis of all 10 studies, including the case-control and cohort studies, found that tea drinking was not associated with laryngeal carcinoma (RR = 1.03; 95% CI: 0.66–1.61). However, coffee consumption was significantly associated with the laryngeal carcinoma (RR = 1.47; 95% CI: 1.03–2.11). A dose-response relationship between coffee intake and laryngeal carcinoma was detected; however, no evidence of dose-response link between tea consumption and laryngeal carcinoma risk was detected.

**Conclusions:**

The results from this meta-analysis of observational studies demonstrate that coffee consumption would increase the laryngeal cancer risk, while tea intake was not associated with risk of laryngeal carcinoma.

## Introduction

It was reported that cancer continues to be a major public health problem despite of the development in medical technology for its prevention, diagnosis and treatment. Cancer of the larynx is fourteenth most common cancer in the world and it is the most common cancer in the head and neck [1]. Tobacco smoking and alcohol drinking are the two major risk factors for the laryngeal carcinoma in the developed countries [2], however, some dietary factors such as vitamins and fiber intake were reported to be protective factors for laryngeal cancer [3]. In a case-control study from Italy and Switzerland, fruit and vegetables diversity is related to a decreased risk of laryngeal cancer risk [4].

Tea and coffee are the most commonly consumed beverages in the worldwide. Numerous studies have been conducted to investigate the association between their consumption and risk of kinds of cancers [5], [6]. Tea, which has attracted much attention for the potential cancer preventive effect for a long time, is reported to be inversely associated several cancer. In the Ohsaki Cohort study involving 41,761 Japanese adults, green tea consumption is associated with a reduced risk of liver cancer incidence [7]. In a cross-sectional study conducted by Il'yasova showed that tea consumption (2–3 and >3 versus <2 servings/day) showed a weak negative association with colorectal adenomas [8]. Coffee is a rich source of various polyphenols. The polyphenols are known to be importart antioxidant properties which are beneficial against several oxidative stress related diseases such as cancer, cardiovascular diseases, and aging [9]. The effect of coffee consumption on the incidence was detected in numerous studies [10] and heterogeneous conclusions were gotten. For instance, a cross-sectional with 93,676 participants showed that daily consumption of six or more cups was associated with a 30% reduced prevalence of nonmelanoma skin cancer. In contrast to caffeinated coffee, daily consumption of decaffeinated coffee was not associated with a significant change in self-reported nonmelanoma skin cancer for Caucasian women [11]. The association between consumption of tea and coffee and risk of laryngeal cancer was reported, however, no consistent conclusion was obtained [12]–[15].

Meta-analysis is a useful statistical tool to pool the relevant studies together and gain a more powerful conclusion. The meta-analysis was also conducted for the searching for the potential causes of laryngeal cancer. For instance, Li X et al investigate the association between human papillomavirus (HPV) infection through combining the relevant studies together and they found that HPV infection, especially infection due to the high-risk type HPV-16, was significantly associated with the risk of laryngeal carcinoma [16]. The aim of this review was to evaluate the evidence from observational studies on tea and coffee consumption on the risk of laryngeal cancer by summarizing it quantitatively with a meta-analysis approach.

## Methods

### Search Strategy and Inclusion Criteria

We followed the Meta-Analysis of Observational Studies in Epidemiology (MOOSE) [17] and Preferred reporting items for systematic reviews and meta-analyses (PRISMA) [18] guidelines in conducting this meta-analysis. A systematic literature search was conducted through two electronic databases (Medline and EMBASE) until Oct. 2013. The key words “tea”, “green tea”, “black tea”, “coffee”, “caffeine”, “beverages”, “diet” and “laryngeal cancer”, “laryngeal carcinoma” were searched as text word and exploded as medical subject headings (MeSH) where possible. The reference lists of relevant articles were reviewed for the additional studies. No language or other restrictions were set in the literature search or the inclusion criteria. If additional data was required, the corresponding authors will be contacted.

The studies were be considered included if they met the following inclusion criteria: 1) studies reported the association between tea or coffee or both and risk of laryngeal cancer; 2) studies obtained a case–control or cohort study design; 3) the value of relative risk (RR), odds ratio (OR) with 95% confidence intervals (CI) or the raw data to calculate them were reported [13], [19], [20].

### Data Extraction and Assessment of Study Quality

The data extraction was conducted via a standardized data extraction form, collecting information on the name of first author, the publication year, study design, number of cases and controls, sample size, study site, gender of participants, type of the tea, tea drinking definitions, adjustments of the confounding factors, and the OR/RR value with 95% CI. When the tea drinking wasn't stated, it would be categorized as “ever versus never”. When the OR or RR was not reported in the article, the RR with 95% confidence intervals (CI) with the raw data and no confounding factors were adjusted. If only stratified results (e.g., by tea or coffee dose) were provided, fixed-effect methods were obtained to summarize the results into a single parameter for each study.

The study quality was assessed by two reviewers back to back and any discrepancies were re solved by reevaluating the included articles and discussion with a third investigator. We obtained the Newcastle-Ottawa Scale (NOS) Assessment of the quality of the included studies. The study quality was assigned to each study based on the 3 parts: selection, comparability, and exposure and outcome condition. The NOS assessed the selection, comparability and exposure of a case-control study, while the selection, comparability and outcome of a cohort study. The study with more than 6 stars would be regarded in relative high quality.

### Data Integration and Statistical Methods

The RR was obtained to approximate RR in this meta-analysis because of the low incidence rate of laryngeal cancer. When both the crude and the adjusted OR/RR values were offered, only the adjusted value would be adopted for the meta-analysis. If only the raw data was reported, we would calculate the unadjusted RR.

The ORs and 95% CIs of all the included studies were pooled using the general variance-based method with a random-effects model. The heterogeneity among the included studies was measured by the χ^2^ test and quantified with the I^2^ statistic. When *P* for the heterogeneity was <0.1 and *I^2^*>50%, the interstudy heterogeneity would be considered statistically significant.

The source of the statistically significant heterogeneity was assessed by both removing the included studies one by one to measure whether any single study was the source of the heterogeneity. Another independent method to detect the source of heterogeneity was to conduct a subgroup meta-analysis. Subgroup analyses were conducted by the study designs (case-control or cohort study), population of or hospital based design, prospective or retrospective study design, and the different sexual groups (male and female group).

We examined a potential linear dose-response relationship between drinks consumption (both tea and coffee consumption were considered) and risk of laryngeal carcinoma using restricted cubic splines with three knots at percentiles 25%, 50%, and 75%of the distribution. A two-stage random-effects dose-response meta-analysis tested was adopted for the detection of the potential nonlinear relation. A P value for nonlinearity was calculated by testing the null hypothesis that the coefficient of the second spline is equal to 0 [21].

A sensitivity analysis was performed by excluding the studies with a relative lower methodological quality. We would assess the effect of tea and coffee consumption and risk of laryngeal carcinoma through just including the high-quality studies. The publication bias was evaluated using funnel plots and the Egger test [22], [23]. P<0.1 was considered to indicate statistically significant publication bias All analyses were conducted using STATA software, version 10.0 (StataCorp LP, College Station, Texas).

## Results

### Identification and Selection of Studies

**Figure 1:**
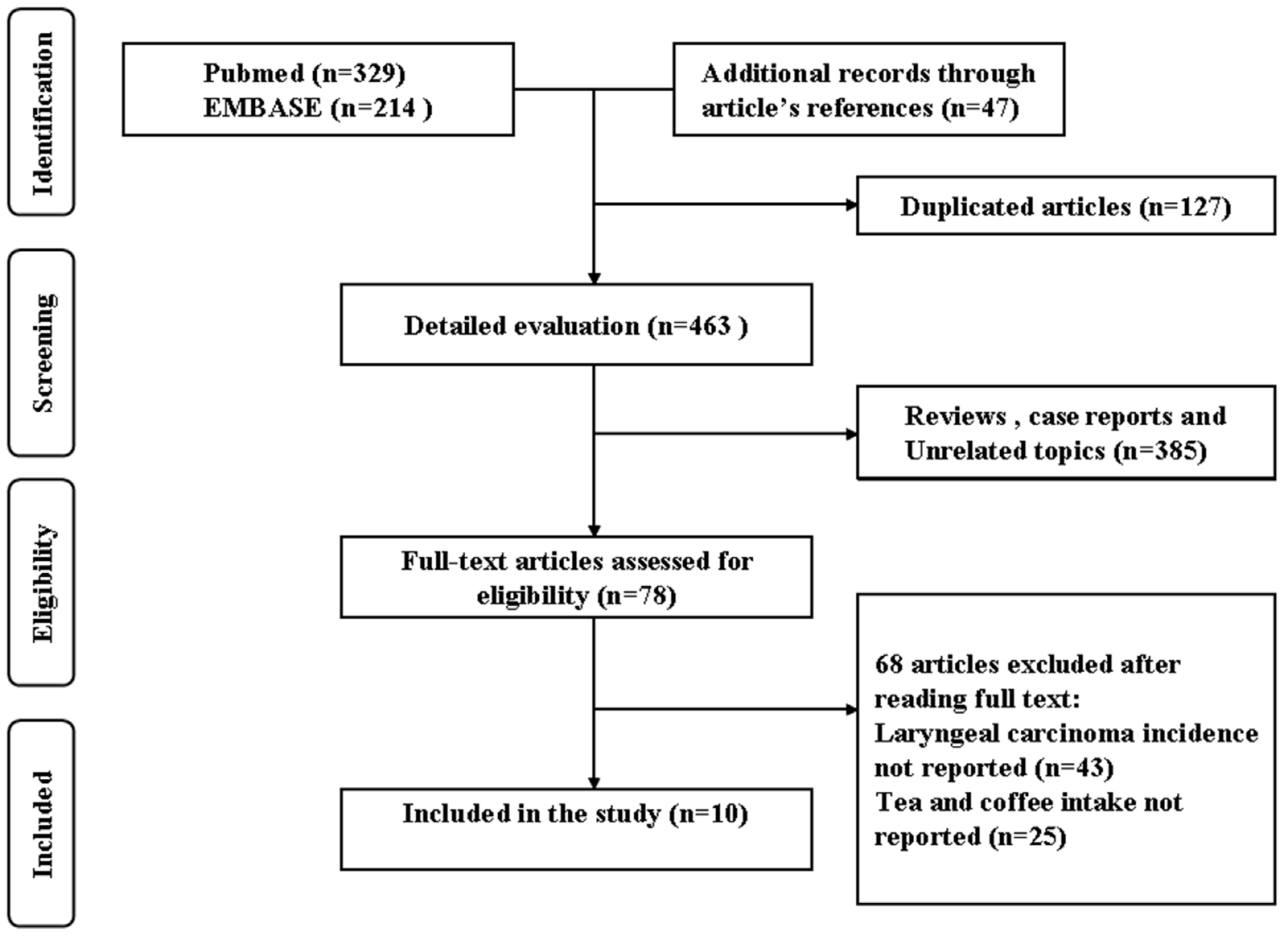
Flow diagram of screened, excluded, and analysed publications.

The flowchart of the study selection was presented in Fig. 1. A total of 801 publications were retrieved from the initial literature search (329 form the Medline, 214 from the EMBASE, and 47 from the reference lists of the relevant studies). After excluding 127 duplicated articles, a total of 463 records were detailed evaluated. Among the 463 articles, 78 full-texts were assessed for eligibility after removing 385 articles (reviews, case reports and unrelated articles). From these, 10 original articles that included data on the association between tea and coffee consumption and laryngeal cancer were ultimately included in our meta-analysis [12]–[15], [19], [20], [24]–[27]. The 68 articles that excluded after reading the full text were the studies in which laryngeal carcinoma incidence not reported (n = 43) and tea and coffee intake not reported (n = 25).

### Study Characteristics and Quality

A total of 2,803 cases and 503,234 controls in 10 independent studies were identified. Among the 10 included studies, 3 reported the data of tea consumption, 3 reported coffee consumption, 3 reported both tea and coffee consumption and 1 pool the data of tea and coffee together. Overall, one study was a prospective population based cohort study and the rest 9 studies were retrospective hospital based case-control studies. The geographicical distribution of the studies sties was 6 were in European, 2 in Americas and 2 in Asia. The detailed age, gender distribution, categories of tea and consumption, and adjustments of confounding factors were demonstrated in Table 1.

**Table 1:** Characteristics of eligible studies.

	Author year	Study design	Site	Age (Year)	No. of cases	No. of cohort/control	Adjusted/Matched factors	Gender (Percent)	Exposure Definition
	Notani PN, et al 1987 [16]	Hospital/Population based Case—control	India	<40∼>60	80	Hospital 215; Population 177	Age and tobacco habits	M (100%)	2,>2 cups/day vs. <2 cups/day
	Pintos J, et al 1994 [17]	Hospital based Case—control	Brazil	<54∼>75	97	756	Tobacco, alcohol, income, rural residency, 10 dietary variables and consumption of other nonalcohol beverages	M (90.74%)	3, Never, ≤1 cup/d, ≥2 cups/d,
Tea	Ren JS, et al 2010 [18]	Population based Cohort	USA	50–71	179	484385	age, sex, tobacco smoking, alcohol drinking, BMI, education, ethnicity, usual physical activity throughout the day, vigorous physical activity and the daily intake of fruit, vegetables, red meat, white meat and calories	M (59.97%)	5, None, <1 cup/M, 1–3 cups/M 1–6 cups/W, ≥1 cup/d
	Galeone C, et al 2010 [19]	Case—control	Europe and USA	18–80	1178	8931	age, sex, race/ethnicity, education, study, cigarette smoking (pack-years), duration of cigar smoking, duration of pipe smoking, alcohol intake, weight, and vegetable and fruit intake.	M (76%)	3, Never,≤1 cup/d,>1 cup/d
	La Vecchia C, et al 1992 [20]	Population based Case—control	Italy	<85	149	6,147	age, sex, area of residence, education, smoking, and coffee consumption	NA	2, Nondrinker, drinker
	Kapil U, et al 2005 [21]	Hospital based Case—control	India	41–80	305	305	NA	M (91.80%)	2, Nondrinker, drinker
	Vassileiou A, et al 2012 [22]	Hospital based Case—control	Greece	<80	70	70	Age, sex	NA	2, Nondrinker, drinker
	Zvrko E, et al 2008 [23]	Hospital based Case—control	Montenegro	35–85	108	108	Age, sex, residence, alcohol, smoking and other risk factors not reported	M (86%)	3 Never,>2 cups/d, >5 cups/d
	La Vecchia C, et al 1990 [24]	Hospital based Case—control	Italy	<45∼74	110	843	Age	M	3, Low, intermediate, high
Coffee	Pintos J, et al 1994 [17]	Hospital based Case—control	Brizil	<54∼>75	97	756	Tobacco, alcohol, income, rural residency, 10 dietary variables and consumption of other non alcohol beverages	M (90.74%)	4, ≤1 cups/d, 1–2 cups/d, 2–3 cups/d, ≥4 cups/d
	Ren JS, et al 2010 [18]	Population based Cohort	USA	50–71	179	484385	Age, sex, tobacco smoking, alcohol drinking, BMI, education, ethnicity, usual physical activity throughout the day, vigorous physical activity and the daily intake of fruit, vegetables, red meat, white meat and calories	M (59.97%)	None, <1 cup/M, 1–3 cups/M 1–6 cups/W, ≥1 cup/d
	Galeone C, et al 2010 [19]	Case—control	Europe and USA	18–80	1178	8931	age, sex, race/ethnicity, education, study, cigarette smoking (pack-years), duration of cigar smoking, duration of pipe smoking, alcohol intake, weight, and vegetable and fruit intake.	M (76%)	,3 Never,≤1,>1
Tea+ Coffee	Bosetti C, et al 2002 [25]	Hospital based Case—control	Italy and Switzerland	30–79	527	1297	Age, sex, center, education, smoking, alcohol, nonalcoholic energy	M (83.88%)	2, Nondrinker, drinker

NA: not available; BMI: body-mass index; M: male; m: month; w: week; d: day.

Study quality was judged on the basis of the Newcastle-Ottawa Scale (1–9 stars). The scale distribution was from 5 to 8 stars. Among the 10 included studies, 8 studies demonstrated a relatively high quality (more than 6 stars in NOS) (Table 2).

**Table 2:** Quality assessment of included studies^1^.

		Quality assessment criteria			
Author	Study design	Selection	Comparability	Outcome/exposure	Overall quality
Notani PN, et al [16]	Case-control	**	**	*	5
Pintos J, et al [17]	Case-control	***	*	**	6
Ren JS, et al [18]	Cohort	***	***	**	8
Carlotta Galeone, et al [19]	Case-control	***	***	**	8
La Vecchia C, et al [20]	Case-control	**	*	**	6
Kapil U, et al [21]	Case-control	**	**	**	5
Vassileiou A, et al [22]	Case-control	**	**	**	6
Zvrko E, et al [23]	Case-control	***	**	**	7
La Vecchia C, et al [24]	Case-control	***	***	**	8
Bosetti C, et al [25]	Case-control	***	**	**	7

1 The quality of studies were assessed by the NOS scale.

### Tea and Coffee Consumption and Laryngeal Cancer

**Figure 2:**
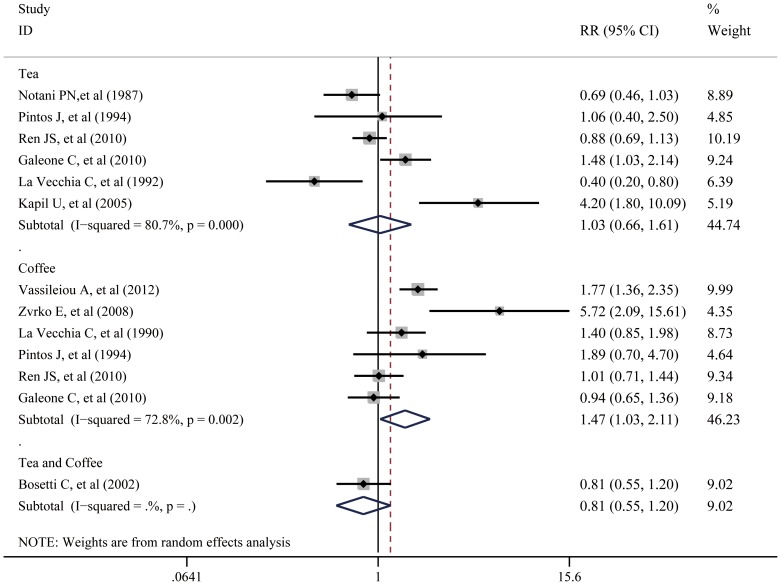
Forest plot: overall meta-analysis of coffee, tea or combined consumption and laryngeal cancer risk. A random-effects model was obtained. Squares indicated study-specific risk estimates (size of square reflects the study-statistical weight, i.e. inverse of variance); horizontal lines indicate 95% confidence intervals; diamond indicates summary relative risk estimate with its corresponding 95% confidence interval.

The overall analysis of all 10 studies, including the case-control and cohort studies, found that tea drinking was not associated with laryngeal carcinoma (RR = 1.03; 95% CI: 0.66–1.61). However, coffee consumption was significantly associated with the laryngeal carcinoma (RR = 1.47; 95% CI: 1.03–2.11). When the relationship between tea and coffee consumption and risk of laryngeal carcinoma was considered, no significant association was detected in the meta-analysis (RR = 1.20; 95% CI = 0.92–1.56) nor the subgroup analyses (RR = 0.81; 95% CI = 0.55–1.20) (Fig. 2).

The subgroup analyses were conducted by the study designs, population or hospital based design, prospective or retrospective design, study sites and the tea drinking category. The effects of tea and coffee consumption and laryngeal carcinoma were detected discretely in the subgroup analyses. Tea consumption was not associated with laryngeal cancer in neither cohort (RR = 0.88, 95% CI = 0.69–1.13) nor case-control studies (RR = 1.09; 95% CI = 0.57–2.08). The similar results were detected in the subgroup analyses by the detailed study designs and the study site. When comparing the one who ever drank tea with never drank tea, tea consumption was unrelated with the laryngeal carcinoma (RR = 1.06; 95% CI = 0.62–1.81). No association was detected when the moderate with the low ranks of tea drinking (RR = 0.97; 95% CI = 0.79–1.20) (Table 3).

**Table 3:** Subgroup analysis of tea and coffee consumption and risk of laryngeal cancer.

	Subgroups	Tea					Coffee				
		Summary Effect			Study Heterogeneity		Summary Effect			Study Heterogeneity	
		RR	(95% CI)	P Value	I^2^, %	p Value	RR	(95% CI)	p Value	I^2^, %	p Value
	Prospective Cohort	0.88	0.69 to 1.13	0.327	-	-	1.01	0.71 to 1.44	0.956	72.7	0.1466
Study design	Retrospective Case-control	1.09	0.57 to 2.08	0.665	84.0	<0.001	1.64	1.08 to 2.50	0.021	-	-
	Population	0.64	0.30 to 1.37	0.123	77.6	<0.001	1.01	0.71 to 1.44	0.956	72.7	0.001
Data source	Hospital	1.38	0.71 to 2.68	0.345	82.1	0.001	1.64	1.08 to 2.50	0.021	-	-
	Europe	0.94	0.39 to 2.28	0.892	90.7	0.001	1.625	1.01 to 2.61	0.045	79.2	0.002
Site	Americas	0.89	0.71 to 1.14	0.361	0	0.708	1.175	0.70 to 1.99	0.548	31.6	0.227
	Asia	1.63	0.28 to 9.60	0.591	92.8	<0.001	-	-	-	-	-
Comparison	Ever vs Never	1.06	0.62 to 1.81	0.837	83.5	0	1.361	1.13 to 1.64	0.001	81.6	0.004
	Moderate vs Low	0.97	0.79 to 1.20	0.789	35.8	0.21	1.251	0.914–1.71	0.162	56.5	0.056

Among the 6 studies that reported the association between coffee consumption and laryngeal carcinoma, 1 study was a prospective population based cohort study, while the rest 5 studies were retrospective hospital based case-control studies. The subgroup analyses of the prospective population based cohort study demonstrated that coffee intake wasn't associated with the laryngeal carcinoma (RR = 1.01; RR = 0.71–1.44). However, this result should be considered with caution considering that only one study was included in this subgroup. The rest 5 study were pool as the retrospective, hospital based and case-control subgroups and a significant association was detected (RR = 1.64; RR = 1.08–2.50). In the subgroup analyses by the study site, a significant result was detected in the Europe subgroup (RR = 1.63; 95% CI = 1.01–2.61) but not in the Americas (RR = 1.18; 95% CI = 0.70–1.99). When the ever coffee drinker was compared with the never drinker, ever coffee drinking was associated with the incidence of laryngeal carcinoma (RR = 1.36; 95% CI = 1.13–1.64). No statistically significant association was detected in the comparison between moderate and low coffee drinking (RR = 1.25; 95% CI = 0.91–1.71).

### Test for the Heterogeneity

The heterogeneity was statistically significant when all the studies were pooled together (I^2^ = 79.8%; P<0.001). The significant heterogeneity was detected when the association between tea (I^2^ = 81.1%; P<0.001) and coffee consumption (I^2^ = 72.8%; P = 0.002) and the laryngeal carcinoma was tested. We tried to explore the source by excluding the included studies one by one and re-count the heterogeneity and the analyses did not identify any one study which contributed significantly to the heterogeneity. The advanced subgroup analyses by study designs, study sites, and case group definitions demonstrated no satisfactory results in the exploring the source of the significant heterogeneity. In advanced studies, the meta-regression showed no satisfactory results in this meta-analysis.

### Dose-Response Meta-Analysis

**Figure 3:**
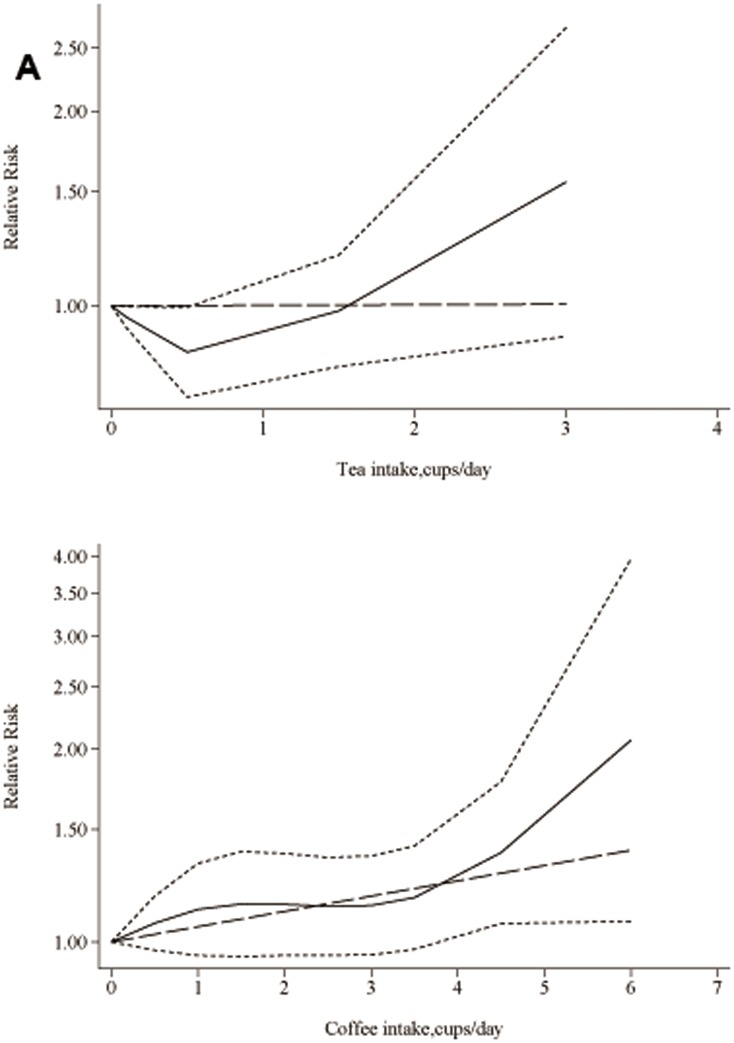
Dose-response relation between tea and coffee consumption and relative risks of laryngeal cancer. A) Dose of tea intake and risks of laryngeal cancer; B) Dose of coffee intake and risks of laryngeal cancer. Lines with short dashes represent the pointwise 95% confidence intervals for the fitted nonlinear trend (solid line). Lines with long dashes represent the linear trend.

Then, we assessed the dose-response relationship between tea and coffee consumption and the risk of laryngeal cancer. We found obvious evidence of statistically significant association of dose of coffee consumption and risk of laryneagl (P = 0.001). A 1 pack of coffee consumption increment conferred a RR of 1.22 (95%CI, 1.04–1.54; Fig 3b) [10]–[12], [17]. However, when tea consumption was considered, no evidence of dose-response relationship was detected (P = 0.342) [10]–[12].

### Sensitivity Analysis and Publication Bias

When only the articles with a relative high quality (over 6 stars NOS stars) included in the meta-analysis, the results was notablely influenced. In the sensitivity analysis, the tea was not associated with laryngeal carcinoma (RR = 0.93; 95% CI: 0.63–1.39), while coffee drinking increased the risk of laryngeal carcinoma (RR = 1.47; 95% CI: 1.03–2.11). A significant heterogeneity should be noted as well (tea: I^2^ = 76.0%, P = 0.006; coffee: I^2^ = 72.8%, P = 0.002).

**Figure 4:**
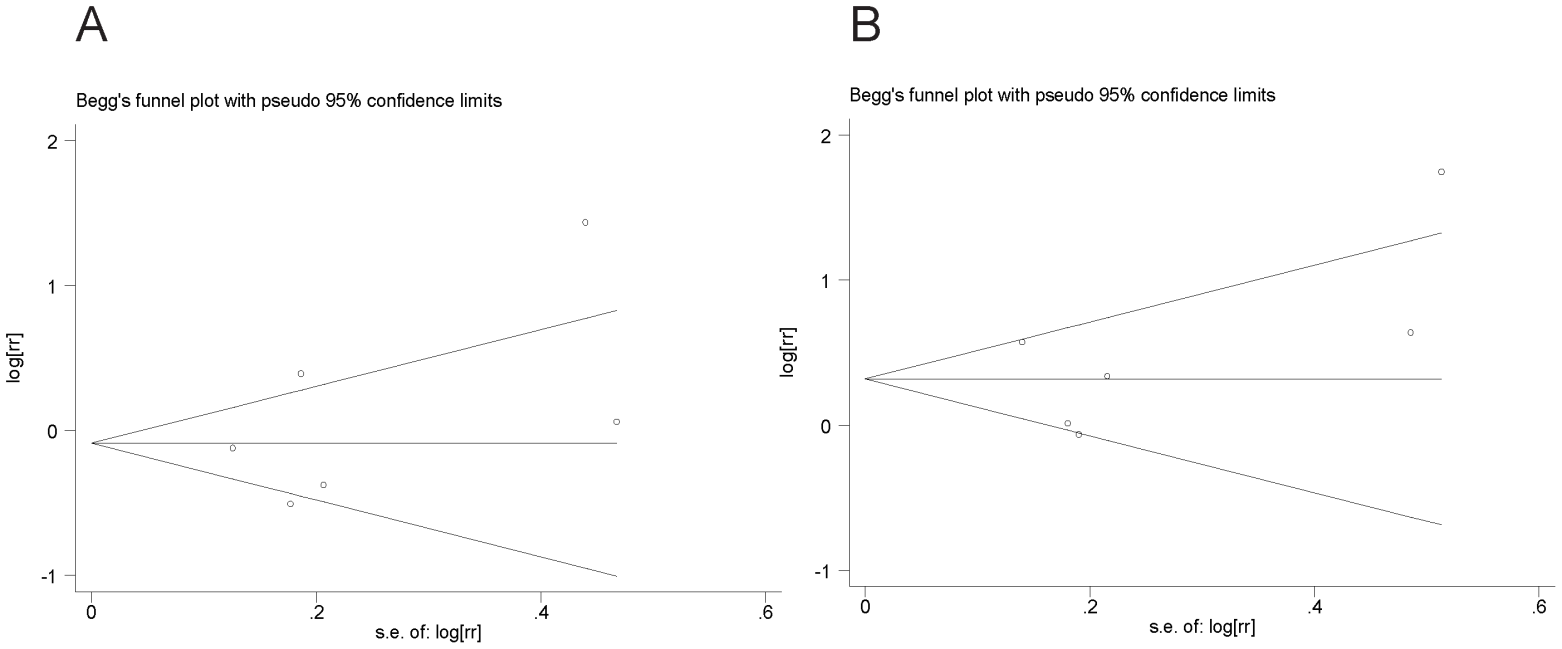
Funnel plot of all the included studies. Funnel plot of the RR vs the standard error of the log RR for studies evaluating tea consumption and laryngeal cancer (A). Funnel plot of the RR vs the standard error of the log RR for studies evaluating tea consumption and laryngeal cancer (B).

The funnel plot for both tea intake (Fig. 4A) and coffee intake (Fig. 4B) and risk of laryngeal carcinoma. No indication of publication bias was observed in the literature on tea (Egger's test, *P* = 0.352) and coffee consumption (Egger's test, *P* = 0.446) and risk of laryngeal carcinoma.

## Discussion

In this current meta-analysis, a total of 2,803 cases and 503,234 controls in 10 independent studies were identified. The quantitative synthesis of these observational studies demonstrated that tea consumption was not associated the incidence of laryngeal carcinoma, while coffee consumption was associated the increased the risk of laryngeal cancer. When the association between tea consumption and risk of laryngeal cancer was considered, the subgroup analysis by the study designs, study sites and tea drinking category displayed no statistical significant results. However, when the association between tea consumption and risk of laryngeal cancer was considered, coffee consumption reduced the risk of laryngeal carcinoma in the retrospective hospital based case-control studies, while no association was detected in prospective population based cohort study. When the ever coffee drinker was compared with the never drinker, ever coffee drinking was associated with the incidence of laryngeal carcinoma. No statistically significant association was detected in the comparison between moderate and low coffee drinking. The heterogeneity was statistically significant when all the studies were pooled together, while no satisfactory results was demonstrated in the exploring the source of the significant heterogeneity. The results of the sensitivity analyses suggest that the conclusion was quite robust.

The etiology of laryngeal cancer is quite unclear by now. In general, it is a disease caused by both genetic and environmental factors. By now, several genetically polymorphic enzymes like cytochrome P450 1A1 are reported to be related with laryngeal cancer [28]. Besides, several other kinds of environmental factors, such as alcohol intake [29], human papillomaviruses infection [30] and silica exposure [31] are also reported to be associated with risk of laryngeal carcinoma. Tea is regarded as a proactive factor of several kinds of cancer [32]. In this meta-analysis, tea consumption wasn't associated with the laryngeal cancer risk. In the advanced stratifying analyses, no more significant results were obtained. Even a significant heterogeneity was detected; however, it was easy to understand considering the existing heterogeneity in the study designs and data set. This result was similar with most previous studies. For instance, a matched case-control study conducted in Southern Brazil showed that tea drinking was not associated with laryngeal cancer [14]. While in a case-control studies conducted in northern Italy between 1983 and 1990, tea consumption was inversely associated with the laryngeal cancer incidence after allowance for age, sex, area of residence, education, smoking, and coffee consumption [24]. Tea drinking was also reported to be a risk factor of the laryngeal cancer in hospital based matched case-control without adjusting for any confounding factors [15]. The sensitivity analysis was conducted through excluding the studies with a lower quality which would produce more potential bias. The sensitivity analyses suggest that no evidence existed among the association between tea drinking and laryngeal cancer risk.

Several previous meta-analyses have been conducted to explore the association between coffee consumption and cancer risk. Both a protective and a harmful effect of tea drinking were detected on the incidence of cancer were detected according to the cancer types [33], [34]. In this meta-analysis, coffee consumption demonstrated as a risk factor of the laryngeal carcinoma. Among all the included studies, there is not any study reporting that coffee consumption increased the risk of laryngeal cancer. This significant association was supported by several independent studies. In a case-control study including 70 patients with laryngeal cancer and 70 controls with non-neoplastic conditions, coffee consumption was reported to be significantly associated with the increased risk of laryngeal cancer [25]. Zvrko E et al conducted a hospital-based case-control study to identify the possible risk factors of the laryngeal carcinoma [26] and they found that coffee consumption more than 5 cups per day was a significant risk factor. Besides, a significant association between coffee consumption and risk of upper digestive and respiratory tracts was detected in a comprehensive meta-analysis [35]. Through pooling 3 relevant studies, it was reported that coffee consumption was not associated the cancer of larynx. However, that meta-analysis included only the observational studies published before October 2009 and only a limited number of studies were included. Through accessing more relevant studies, this meta-analysis obtained an updated conclusion. In the advanced subgroup analyses by the tea drinking category, more abundant results are reported. Comparing the ever coffee drinker with the never drinker, ever coffee drinking was associated with an increased incidence of laryngeal carcinoma. However, no statistically significant association was detected in the comparison between moderate and low coffee drinking. It suggested that moderate coffee might be unrelated with the increased risk of laryngeal cancer, however, this conclusion should be considered with great cautious.

Tea and coffee are served as hot beverage usually. They might modify the incidence of laryngeal cancer through more than the tea and coffee itself. In a systematic review, the results strongly suggest that high-temperature beverage drinking increases the risk of esophageal cancer [36]. We speculate that the hot temperature would decline the potential protective effects of tea and coffee. Ren JS et al conducted a prospective cohort study, the effects of both iced and hot tea on the laryngeal cancer incidence were investigated [13]. Neither the iced tea (HR = 0.86; 95% CI = 0.62–1.18) or hot tea (HR = 0.92; 95% CI: 0.63–1.36) consumption was reported to be associated the incidence of laryngeal cancer after adjusting the relevant confounding factors, such as age, sex, tobacco smoking, alcohol drinking, BMI, education and ethnicity. The temperature of tea and coffee is a potential important factor in their effects on the laryngeal cancer risk.

There are no definite biological mechanisms of the potential harmful role of coffee on laryngeal cancer, thus there are still no explanations for the different effects of tea and coffee on laryngeal cancers. In particular, the coffee drinks contains several phenolic compounds (such as chlorogenic, caffeic, and cumaric acids), melanoidins and diterpenes, which might produce certain effects on the development of laryngeal cancer [37].

To our best knowledge, this is the first meta-analysis investigating the relationship between tea consumption and risk of laryngeal cancer. A comprehensive literature search and advanced detailed consulting the relevant references was conducted to make sure all the potential logical articles included. In this current meta-analysis, the results of the sensitivity analyses suggest that the conclusions of this study were quite robust. The stratified analyses and detailed dose-response analysis provided abundant knowledge.

Despite these strengths mentioned above, some limitations of the current meta-analysis should be noted. Firstly, only 10 studies with relative low quality were included in this meta-analysis and the relative small sample size would make the conclusion unstable. We have tried our best to access all the possible studies. In the quantitative synthesis, the random-effect model might provide a more conservative conclusion. Secondly, the most of our studies followed a case–control study design, and therefore there were recall and selection bias which are inherent to retrospective studies. More relevant cohort studies are required in the future. Besides, considering the significance of between-study heterogeneity, the conclusions should be considered with caution.

In conclusion, tea consumption wasn't associated with the risk of laryngeal cancer, while coffee consumption increased the laryngeal cancer risk. Nevertheless, because of the potential limitations of this meta-analysis, conclusions must be drawn with caution, and more well-designed studies with large sample sizes should be conducted for further validation.

## Supporting Information

S1 Checklist PRISMA checklist.
(DOC)
